# A multicenter prospective study on postoperative pulmonary complications prediction in geriatric patients with deep neural network model

**DOI:** 10.3389/fsurg.2022.976536

**Published:** 2022-08-09

**Authors:** Xiran Peng, Tao Zhu, Guo Chen, Yaqiang Wang, Xuechao Hao

**Affiliations:** ^1^ Department of Anesthesiology, National Clinical Research Center for Geriatrics, West China Hospital, Sichuan University, Chengdu China; ^2^ The Research Units of West China (2018RU012) -Chinese Academy of Medical Sciences, West China Hospital, Sichuan University, Chengdu China; ^3^ College of Software Engineering, Chengdu University of Information Technology, Chengdu China

**Keywords:** postoperative pulmonary complications, deep neural network model, geriatric assessment (MeSH), risk assessment, electronic health records

## Abstract

**Aim:**

Postoperative pulmonary complications (PPCs) can increase the risk of postoperative mortality, and the geriatric population has high incidence of PPCs. Early identification of high-risk geriatric patients is of great value for clinical decision making and prognosis improvement. Existing prediction models are based purely on structured data, and they lack predictive accuracy in geriatric patients. We aimed to develop and validate a deep neural network model based on combined natural language data and structured data for improving the prediction of PPCs in geriatric patients.

**Methods:**

We consecutively enrolled patients aged ≥65 years who underwent surgery under general anesthesia at seven hospitals in China. Data from the West China Hospital of Sichuan University were used as the derivation dataset, and a deep neural network model was developed based on combined natural language data and structured data. Data from the six other hospitals were combined for external validation.

**Results:**

The derivation dataset included 12,240 geriatric patients, and 1949(15.9%) patients developed PPCs. Our deep neural network model outperformed other machine learning models with an area under the precision-recall curve (AUPRC) of 0.657(95% confidence interval [CI], 0.655–0.658) and an area under the receiver operating characteristic curve (AUROC) of 0.884(95% CI, 0.883–0.885). The external dataset included 7579 patients, and 776(10.2%) patients developed PPCs. In external validation, the AUPRC was 0.632(95%CI, 0.632–0.633) and the AUROC was 0.889(95%CI, 0.888–0.889).

**Conclusions:**

This study indicated that the deep neural network model based on combined natural language data and structured data could improve the prediction of PPCs in geriatric patients.

## Introduction

More than 300 million surgeries are performed worldwide each year (1). Around one-third of elective surgeries are performed on patients aged over 65 years (2). Compared with younger adults, older individuals are more prone to postoperative complications because of age-related degenerative physiological characteristics (3).

Postoperative pulmonary complications (PPCs), including respiratory infection, atelectasis, and respiratory failure, are common, and even mild PPCs are associated with a prolonged hospital stay and increased postoperative mortality (4–6). The incidence of PPCs in major surgery ranges from 1% to 23% depending on different PPCs definitions and surgical specialties (7), and the postoperative mortality rate of patients with PPCs varies from 14% to 48% (8–10). Hospital stay is prolonged by 13–17 days in patients with PPCs (7). For the management of PPCs, preventive strategies may be more effective than treating established PPCs (11). Preoperatively identifying the risk of PPCs is critical for guiding preventive interventions to reduce the risk and incidence of PPCs (12).

Most risk assessment tools for PPCs were developed using traditional logistic regression (13), such as the Assess Respiratory Risk in Surgical Patients in Catalonia (ARISCAT) risk score (14). Traditional logistic regression constrains the number of input risk factors, which may omit potential predictors and limit the predictive accuracy (15). Machine learning algorithms are advantageous in that they can identify hidden insights from large datasets (16), which can help to build more accurate prediction models for PPCs (13).

Recent studies (17–19) have demonstrated the superiority of machine learning algorithms in predicting PPCs. For example, Xue et al. (19) used structured perioperative data to predict five postoperative complications, including pneumonia. To further improve predictive performance, overcoming several methodological deficiencies may be effective. First, most models are based purely on structured data, and natural language data are not generally included. Underutilizing natural language data in model development may cause loss of clinical information and limit predictive accuracy (20). Second, few predictive models have been developed specifically for geriatric patients. Geriatric patients are at a high risk of developing PPCs (9). Considering age-related physiological characteristics, predictive models based on data from the general patient population may be unsuitable for geriatric patients (21). Third, most studies lack external validation; thus, it is uncertain whether existing models could achieve comparable predictive performance at other institutions (13).

In this study, we aimed to develop and validate a deep neural network model to predict PPCs in geriatric patients based on combined natural language data and structured data. We hypothesized that this model could accurately predict patients who are at a high risk of developing PPCs.

## Method

### Data source

This study followed the Transparent Reporting of a Multivariable Prediction Model for Individual Prognosis or Diagnosis (TRIPOD) guidelines (22). The study protocol was approved by the ethics committee of the West China Hospital of Sichuan University (2019-473) with a waiver of informed consent. The study is registered at www.chictr.org.cn (ChiCTR1900025160). In this study, we prospectively collected data from seven hospitals in China, including the West China Hospital of Sichuan University, the Second Affiliated Hospital of Chongqing Medical University, the Wuhan Union Hospital, the Guangdong Provincial People's Hospital, the First Affiliated Hospital of Kunming Medical University, the First People's Hospital of Zhaoqing, and the Qingyuan People's Hospital. Patients aged ≥65 years who underwent surgery under general anesthesia between 25th June 2019 and 31st December 2021 were enrolled. If patients underwent multiple surgeries during the study period, only the first surgery was included in the analysis. Related patient data were collected by trained residents on the day before surgery. The attending physician and the resident would re-check the collected information before surgery. If any errors or omissions existed, the clinician would make corrections or supplement the information. Preoperative laboratory tests were automatically retrieved from the laboratory information system. All laboratory tests were performed within 7 days before surgery. If a patient had more than one result for the same test, the most recent preoperative result was used in the analysis. Preoperative clinical data included demographic characteristics, preoperative vital signs, laboratory tests and comorbidities. Supplementary Table S1 shows the 127 variables included in our study.

### Postoperative follow-up

To ascertain the presence of PPCs, we conducted prospective patient follow-up. Research personnel performed follow-up with patients at different time points, including 24 h after surgery, 48 h after surgery, before hospital discharge, and on the 30th day after surgery. If a patient developed PPCs, we stayed in contact with the patient until recovery or death. Throughout each patient's hospital stay, the research personnel conducted bedside follow-up visits, and after hospital discharge, patients were contacted *via* telephone.

### Outcome definition

The outcome was the incidence of any PPC within 30 days after surgery. PPCs included unplanned mechanical ventilation, atelectasis, pulmonary congestion, respiratory infection, pleural effusion, pneumothorax, and respiratory failure. Supplementary Table S2 shows the definition of each PPC.

### Data preprocessing and model development

Variables are presented as numerical, categorical, or free-text variables. Free-text data contain the descriptions of principal diagnoses and comorbidities. Missing values were imputed by 0 s, with indicators representing missingness, which regarded missing values as a separate group. Numerical variables were transformed to categorical variables using 5-bins equal-width scaling. Data from patients admitted to the West China Hospital of Sichuan University were used as the derivation dataset, and a deep neural network model was trained. Five random shuffles of five-fold cross-validation were performed to divide the training set and validation set. In each iteration, a different stratified fold was used for model evaluation, and model training was performed on the remaining folds.

The number of patients without PPCs was much higher than the number of patients with PPCs, which led to class imbalance. Cross-entropy loss function was used to overcome this issue by enhancing the accurate prediction of positive examples. Early stopping and dropout were used to avoid overfitting. Early stopping refers to ceasing model training when the validation loss starts to increase. A patience of 40 epochs was set for early stopping. Dropout is a method to prevent co-adapting by removing neurons from the network (23). In our study, dropout with a probability of 0.1 was applied to all layers.

### Model comparison

Our deep neural network model was compared with several extensively used classifiers, including Elastic Net logistic regression, support vector machine, random forest, gradient boosting machine, and extreme gradient boosting.

To evaluate and compare the different models, we calculated performance metrics using the validation fold in each iteration and took the average over all repetitions. Performance metrics included sensitivity (recall), precision, F1 score, specificity, accuracy, area under the precision-recall curve (AUPRC), and area under the receiver operating characteristic curve (AUROC). Sensitivity reflects the ability to capture positive examples (24). In circumstances with an imbalanced class distribution, the precision and sensitivity can provide more direct insight into predictive performance (25). The F1 score is the harmonic mean of the precision and sensitivity. The AUROC is widely used to estimate the performance of binary classifiers. However, the AUROC can generate misleading conclusions about model performance for classifiers established on imbalanced datasets (26). The AUPRC gives no credit for predicting true negatives, and can provide a more accurate interpretation of a model's actual performance (25). In this study, we chose the F1 score and the AUPRC as the main evaluation metrics for model comparison. The calibration ability was measured using the Hosmer–Lemeshow calibration plot.

The overall architecture of our deep neural network model is depicted in Supplementary Figure S1. To indicate the risk level, we divided patients into three groups with a low, intermediate, and high risk of PPCs based on the predicted probability. The optimal cutoff values were confirmed using the minimum description length principle (MDLP) (27). The chi-square test was performed to compare the incidence of PPCs between the three groups.

The deep neural network model was implemented using PyTorch. Machine learning models were developed in Python 3.8.3 using the scikit-learn library. A *P* value of <0.05 was considered statistically significant.

### Feature importance

In our model, Multi-Head Attention (28) in the Transformer layer could set the weight for each variable. The magnitude of the weight indicates the degree to which the input variable affects the prediction. To gain insight into the workings of our model, we calculated the feature weights for all patients in the derivation dataset using Multi-Head Attention. To illustrate individual risk prediction, we presented two examples and visualized the variable importance in these individual predictions.

### External validation

Data from the other six participating hospitals were combined and used for external validation. These hospitals adopted the same preoperative interview and postoperative follow-up system as the West China Hospital of Sichuan University. We extracted the same features as mentioned above with the exception of some laboratory tests, because we could not retrieve these results from their laboratory information systems. Supplementary Table S1 shows the variables included in the external dataset.

Previous studies indicated the importance of local calibration considering institution-specific differences in patient populations and surgical practices (26, 29, 30). Demonstrating a generalizable method may be more reasonable than developing a globally used predictive model (26). To validate our overall methodology, we applied the same training method to recalibrate the deep neural network model based on the combined external dataset.

## Results

The derivation dataset included 12,240 geriatric patients at the West China Hospital of Sichuan University between 25th June 2019 and 30th April 2021, the majority of whom were men (56.4%). Supplementary Table S3 shows summary statistics for patients' characteristics. Of these patients, 1949(15.9%) patients developed PPCs, including 533(4.4%) with unplanned mechanical ventilation, 526(4.3%) with atelectasis, 32(0.3%) with pulmonary congestion, 1009(8.2%) with respiratory infection, 1267(10.4%) with pleural effusion, 217(1.8%) with pneumothorax, and 163(1.3%) with respiratory failure.

### Comparison of the deep neural network model with other models

**Figure 1:**
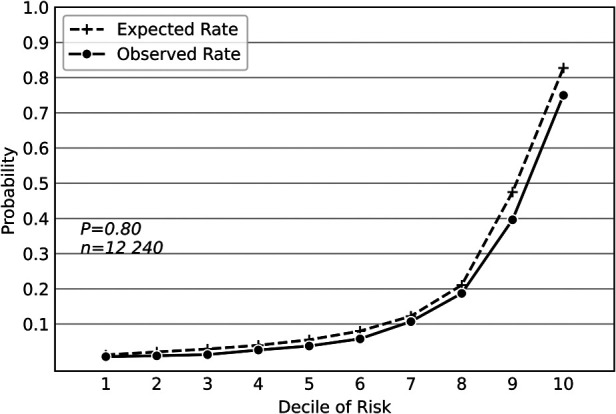
Hosmer-Lemeshow calibration plot of the deep neural network model based on the derivation dataset. Values on the *x*-axis are deciles of predicted risk of postoperative pulmonary complications and values on the y-axis are rates of postoperative pulmonary complications for each decile. The result of Hosmer–Lemeshow test (*P* = 0.80) showed good agreement between the deep neural network model-based prediction and observed outcome.

Compared with other widely used classifiers, the deep neural network model achieved the greatest sensitivity of 0.603(95% confidence interval [CI], 0.602–0.604), the highest F1 score of 0.641(95%CI, 0.640–0.642), the greatest AUPRC value of 0.657(95%CI, 0.655–0.658), and the greatest AUROC value of 0.884(95%CI, 0.883–0.885) (Table 1). Hosmer-Lemeshow calibration plot (*P* = 0.80) showed good agreement between the deep neural network model-based prediction and observed outcome (Figure 1).

**Table 1:** Performance metrics of the deep neural network model and other machine learning models.

Model	Precision (95% CI)	Sensitivity (95% CI)	F1 score (95% CI)	AUPRC (95% CI)	Specificity (95% CI)	Accuracy (95% CI)	AUROC (95% CI)
Extreme gradient boosting	0.675 (0.674–0.676)	0.502 (0.501–0.502)	0.575 (0.575–0.576)	0.605 (0.604–0.605)	0.954 (0.953–0.954)	0.882 (0.881–0.882)	0.866 (0.866–0.867)
Gradient boosting machine	0.687 (0.686–0.687)	0.519 (0.519–0.520)	0.591 (0.591–0.592)	0.620 (0.620–0.621)	0.955 (0.954–0.955)	0.885 (0.885–0.886)	0.878 (0.878–0.879)
Random Forest	0.695 (0.687–0.698)	0.513 (0.512–0.515)	0.590 (0.589–0.592)	0.628 (0.621–0.630)	0.955 (0.954–0.955)	0.886 (0.885–0.886)	0.870 (0.868–0.871)
Support vector machine	0.704 (0.703–0.705)	0.208 (0.207–0.208)	0.321 (0.320–0.322)	0.559 (0.558–0.559)	0.983 (0.983–0.984)	0.860 (0.859–0.860)	0.839 (0.838–839)
Elastic Net logistic regression	0.682 (0.681–0.683)	0.318 (0.318–0.319)	0.434 (0.433–0.434)	0.571 (0.570–0.571)	0.971 (0.971–0.972)	0.867 (0.867–0.868)	0.841 (0.840–0.841)
Deep neural network	0.684 (0.682 –0.686)	**0.603** (0.602–0.604)	**0.641** (0.640–0.642)	**0.657** (0.655–0.658)	0.943 (0.941–0.944)	0.885 (0.884–0.887)	**0.884** (0.883–0.885)
In external validation							
Deep neural network^a^	0.610 (0.609–0.610)	0.596 (0.595–0.596)	**0.602** (0.602–0.603)	**0.632** (0.632–0.633)	0.930 (0.929–0.930)	0.877 (0.877–0.878)	**0.889** (0.888–0.889)

Abbreviations: CI, confidence interval; AUPRC, area under the precision-recall curve; AUROC, area under the receiver operating characteristic curve.

^a^ The deep neural network model retrained on the external dataset.

To indicate the risk level, patients were stratified into three groups with a low, intermediate, and high risk of PPCs based on the risk predicted probability of the deep neural network model. The optimal cutoff values of the risk predicted probability were confirmed by MDLP (27) (low risk: ≤0.3; intermediate risk: 0.3 < risk predicted probability ≤0.7; high risk: >0.7).

The incidence of PPCs was significantly different between the three groups (low-risk group: 5.5%; intermediate-risk group: 42.4%; high-risk group: 76.6%; *P* < 0.001 for all, Supplementary Table S4).

### Feature importance

For patients in the derivation dataset, the top 10 most important variables in the deep neural network model were acidophil count, triglyceride, fibrinogen, functional capacity, platelet count, acidophil percentage, neck movement test, hydroxybutyrate dehydrogenase, mean corpuscular hemoglobin concentration, and mean corpuscular hemoglobin (Table 2).

**Table 2:** Top ten most important variables in the deep neural network model for patients in the derivation dataset and the two case examples.

Patients in the derivation dataset		Patient A		Patient B	
Variable	Importance	Variable	Importance	Variable	Importance
Acidophil count	65.23	Acidophil count	50.89	Triglyceride	50.99
Triglyceride	61.93	Difficult ventilation history	50.02	COPD	50.19
Fibrinogen	60.73	COPD	50.01	Difficult ventilation history	49.98
Functional capacity	59.93	Airway obstruction	49.96	Airway obstruction	49.79
Platelet count	58.78	Respiratory infection within last 1 month	49.70	Free text record	49.48
Acidophil percentage	57.76	Low activity	49.63	Difficult intubation history	49.45
Neck movement test	57.68	Systolic blood pressure	49.45	MCH	49.30
Hydroxybutyrate dehydrogenase	57.52	Free text record	49.23	Upper digestive tract hemorrhage within last 1 week	49.28
MCHC	57.44	Total bilirubin	49.02	NYHA classification	49.27
MCH	57.25	Monocyte percentage	48.94	Decreased endurance	49.09

Abbreviations: COPD, chronic obstructive pulmonary disease; MCH, mean corpuscular hemoglobin; MCHC, mean corpuscular hemoglobin concentration; NYHA, New York Heart Association.

We present two examples to illustrate individual risk prediction. These two patients were in the high-risk group and actually developed PPCs. Table 2 shows the variables that greatly contributed to individual prediction in these two patients. For patient A, the acidophil count contributed the most to risk prediction, with patient A having a high acidophil count (2.10 × 10^9^/L; normal range: 0.02–0.52 × 10^9^/L). For patient B, triglyceride was the most important variable in risk prediction, with patient B having a high triglyceride concentration of 7.35 mmol/L (normal range: 0.29–1.83 mmol/L). Patient B's acidophil count was within the normal range (0.02 × 10^9^/L) and was not important in this prediction, ranking 105th, while patient A's triglyceride concentration was normal (0.94 mmol/L) and was not important in this prediction, ranking 123rd.

### External validation

**Figure 2:**
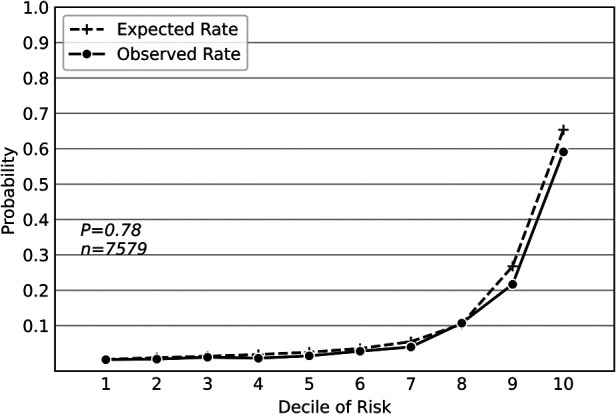
Hosmer-Lemeshow calibration plot of the deep neural network model based on the external dataset. Values on the x-axis are deciles of predicted risk of postoperative pulmonary complications and values on the y-axis are rates of postoperative pulmonary complications for each decile. The result of Hosmer–Lemeshow test (*P* = 0.78) showed good agreement between the deep neural network model-based prediction and observed outcome.

The combined external dataset included 7579 geriatric patients from 23rd April 2021 to 31st December 2021. A total of 776 patients (10.2%) developed PPCs, including 302 (4.0%) with unplanned mechanical ventilation, 240 (3.2%) with atelectasis, 2 (0.03%) with pulmonary congestion, 513 (6.8%) with respiratory infection, 506 (6.7%) with pleural effusion, 4 (0.05%) with pneumothorax, and 112 (1.5%) with respiratory failure. Supplementary Table S5 shows the summary statistics for patients' characteristics in the combined dataset. We applied the same methodology to retrain the deep neural network model based on the external dataset. Although some laboratory tests were not included in the external dataset, the deep neural network model maintained good predictive performance, with an F1 score of 0.602(95% CI, 0.602–0.603), an AUPRC of 0.632(95% CI, 0.632–0.633), and an AUROC of 0.889(95% CI, 0.888–0.889) (Table 1). Hosmer-Lemeshow calibration plot (*P* = 0.78) showed good agreement between the deep neural network model-based prediction and observed outcome (Figure 2).

## Discussion

PPCs are associated with a prolonged hospital stay and increased postoperative mortality (31). Early identification of high-risk patients could help to guide preventive interventions to improve prognosis. This study showed that the deep neural network model based on combined natural language data and structured data could improve the prediction of PPCs in geriatric patients. Patients were stratified into three risk groups to indicate the risk level, and the incidence of PPCs was significantly different among the three groups.

Geriatric patients are at a high risk of developing PPCs (9). In other studies (17–19), the data of older and younger patients have often been pooled together. Considering age-related physiological characteristics, ignoring age categories can cause inaccurate parameter estimation (21) and may decrease the discrimination ability in geriatric patients. Current assessment tools that are based on pooled data often underestimate risk in geriatric patients (21). In this study, we specifically focused on the geriatric population to improve the predictive accuracy in this population specifically.

Deep learning has the advantage of learning directly from natural language data without the need for manual processing (32, 33). In our study, natural language data contained descriptions about principal diagnoses and comorbidities. Considering that International Classification of Diseases codes can only be acquired at discharge, they are not available for preoperative prediction (24), and natural language data could supply this clinical information. In a clinical setting, correctly identifying patients who are at risk of PPCs is critical, so a model with high sensitivity is appropriate (34). Previous studies on predicting PPCs have achieved sensitivities in the range of 0.321–0.526 (4, 18, 19). Compared with previous studies and other models in our study, the deep neural network model achieved the highest sensitivity of 0.603, which indicated that the deep neural network model based on combined natural language data and structured data could more accurately identify patients with PPCs. To process natural language data, we performed embedding using MedBERT and mean-pooling, instead of traditional one-hot encoding. With one-hot encoding, natural language data are actually transformed into binary variables according to the presence or absence of particular words (26), which may hinder the learning of potential relationships between descriptions (32). Embedding does not regard two principal diagnoses as completely different categories. Instead, embedding enable all variables to be present in a multi-dimensional space, and similar features could be mapped next to each other. For example, a cholecystolithiasis is closer to a cholecystolithiasis with chronic cholecystitis than an acute cholecystitis in the embedding space.

In terms of individual predictions, Multi-Head Attention (28) in the deep neural network model was used to set the corresponding weight for each variable according to the patient-specific input value, instead of setting a fixed weight for each variable, as is the case with logistic regression. In the case example, patient A had a high acidophil count, and acidophil count contributed the most to the high risk of PPCs in patient A; thus, it was set the heaviest weight in this prediction. Patient B had a normal acidophil count, but the patient's triglyceride concentration was high and contributed the most to the high risk of PPCs; thus, triglyceride was set the heaviest weight in this individual prediction. Patient-specific characteristics may lead to more accurate individual predictions. After calculating the predictive probability and corresponding risk level, the model could output the variables that contributed to individual prediction. Recognizing these important variables may assist clinicians in early identification of potential factors, which could help to decide the treatment protocol to prevent PPCs and mitigate risk (35).

With ordinary external validation, the model is directly applied to a different dataset. In fact, local-specific parameters in certain models may not be generalizable to other populations considering hospital-specific patient populations and surgical practices (29, 30). Previous studies have emphasized the importance of recalibration to overcome this limitation (26, 29, 30). In our study, we validated the overall modeling methodology by retraining the deep neural network model based on the external dataset. Although some laboratory tests were not included in the external dataset, the recalibrated deep neural network model maintained good predictive performance, which indicated that our methodology was generalizable to other institutions, even if they could not collect complete features.

Our study has several limitations that should be noted. First, although the model could output variables that contributed greatly to individual predictions, the association between important features and outcome was not necessarily causation. We cannot conclude whether guiding the treatment protocol according to these variables could improve prognosis. Further research is necessary to quantify the benefit of this model in guiding interventions and improving patients' outcomes. Second, limited by the small number of patients in each group (divided by surgery type), a subgroup analysis based on the specific surgery type was not conducted. Thus, the model's predictive ability may be limited in some subspecialties.

In conclusion, this study indicated that the prediction of PPCs in geriatric patients could be improved by deep neural network model based on combined natural language data and structured data. The overall modeling methodology was generalizable to other institutions; thus, it could be used to construct their own predictive models.

## Data Availability

The raw data supporting the conclusions of this article will be made available by the authors, without undue reservation.
