# Supplementary material for: A multicenter prospective study on postoperative pulmonary complications prediction in geriatric patients with deep neural network model
Source: Front Surg. 2022 Aug 9;9:976536. doi: 10.3389/fsurg.2022.976536 (PMC9395933; doi:10.3389/fsurg.2022.976536)
Supplement: Supplementary file 1 [file Table_1_v1.docx]

**Supplementary Material**

Supplementary Table S1. The list of variables, missing rates and data type.

| Variable | Missing rate in derivation dataset | Missing rate in external dataset | Data type |
| --- | --- | --- | --- |
| Sex | 0.00% | 0.04% | Categorical |
| Age | 0.00% | 0.01% | Numerical |
| Height | 0.00% | 0.37% | Numerical |
| Weight | 0.00% | 0.30% | Numerical |
| BMI | 0.00% | 0.25% | Numerical |
| Smoking | 0.00% | 0.00% | Categorical |
| Alcohol | 0.00% | 0.00% | Categorical |
| Systolic blood pressure | 0.00% | 0.28% | Numerical |
| Diastolic blood pressure | 0.00% | 0.29% | Numerical |
| Respiratory rate | 0.00% | 0.30% | Numerical |
| Heart rate | 0.00% | 0.30% | Numerical |
| Body temperature | 0.00% | 0.36% | Numerical |
| ASA classification | 0.00% | 0.41% | Categorical |
| NYHA classification | 0.00% | 0.00% | Categorical |
| Functional capacity | 0.00% | 0.00% | Categorical |
| Emergent surgery | 0.00% | 0.00% | Categorical |
| Open surgery | 0.00% | 0.00% | Categorical |
| Endoscopic surgery | 0.00% | 0.00% | Categorical |
| Endovascular surgery | 0.00% | 0.00% | Categorical |
| Surgery type | 0.00% | 0.53% | Categorical |
| Estimated operation time | 0.00% | 0.70% | Categorical |
| Estimated blood loss | 0.00% | 0.73% | Categorical |
| General condition | 0.00% | 0.00% | Categorical |
| Conscious state | 0.00% | 0.00% | Categorical |
| Breath-holding test | 0.00% | 3.87% | Categorical |
| Limited mouth opening | 0.00% | 0.00% | Categorical |
| Mallampati airway classification | 0.00% | 0.00% | Categorical |
| Mandibular protrusion test | 0.00% | 0.00% | Categorical |
| Neck movement test | 0.00% | 0.00% | Categorical |
| Frailty assessment |  |  |  |
| Tiredness | 0.00% | 4.80% | Categorical |
| Decreased endurance | 0.00% | 4.80% | Categorical |
| Low activity | 0.00% | 4.80% | Categorical |
| Comorbidities | 0.00% | 4.80% | Categorical |
| Weight loss | 0.00% | 4.80% | Categorical |
| Comorbidities |  |  |  |
| Hypertension | 0.00% | 0.00% | Categorical |
| Diabetes mellitus | 0.00% | 0.01% | Categorical |
| Respiratory infection within last 1 month | 0.00% | 0.00% | Categorical |
| COPD | 0.00% | 0.00% | Categorical |
| Dyspnea | 0.00% | 0.00% | Categorical |
| Difficult ventilation history | 0.00% | 0.00% | Categorical |
| Difficult intubation history | 0.00% | 0.00% | Categorical |
| Sleep apnea syndrome | 0.00% | 0.00% | Categorical |
| Airway obstruction | 0.00% | 0.00% | Categorical |
| Stroke | 0.00% | 0.00% | Categorical |
| Ischemic heart disease | 0.00% | 0.00% | Categorical |
| Valvular heart disease | 0.00% | 4.80% | Categorical |
| Dilated cardiomyopathy | 0.00% | 0.00% | Categorical |
| Hypertrophic cardiomyopathy | 0.00% | 0.00% | Categorical |
| Congestive heart failure | 0.00% | 0.00% | Categorical |
| Arrhythmia | 0.00% | 0.00% | Categorical |
| Pericardial disease | 0.00% | 0.00% | Categorical |
| Artery disease | 0.00% | 0.00% | Categorical |
| Venous disease | 0.00% | 0.00% | Categorical |
| Lower digestive tract hemorrhage within last 1 week | 0.00% | 0.00% | Categorical |
| Upper digestive tract hemorrhage within last 1 week | 0.00% | 0.00% | Categorical |
| Gastroesophageal reflux | 0.00% | 0.00% | Categorical |
| Peptic ulcer | 0.00% | 0.00% | Categorical |
| Thyroid dysfunction | 0.00% | 0.00% | Categorical |
| Autoimmune disease | 0.00% | 0.00% | Categorical |
| Psychiatric disorder | 0.00% | 0.00% | Categorical |
| Neuromuscular disease | 0.00% | 0.00% | Categorical |
| Preoperative medical treatments |  |  |  |
| Vasoactive medications | 0.00% | 0.00% | Categorical |
| Anti-heart failure drugs | 0.00% | 0.00% | Categorical |
| Anticoagulants and antiplatelet agents | 0.00% | 0.00% | Categorical |
| Glucocorticoid | 0.00% | 0.00% | Categorical |
| Dialysis | 0.00% | 0.00% | Categorical |
| Laboratory tests |  |  |  |
| SpO_2_ | 0.86% | 0.58% | Numerical |
| CO_2_CP | 3.01% | 100% | Numerical |
| Hemoglobin | 2.39% | 1.72% | Numerical |
| Red blood cell count | 2.74% | 100% | Numerical |
| RDW-CV | 2.86% | 100% | Numerical |
| RDW-SD | 2.86% | 100% | Numerical |
| Mean corpuscular hemoglobin | 2.74% | 100% | Numerical |
| MCHC | 2.74% | 100% | Numerical |
| Mean corpuscular volume | 2.74% | 100% | Numerical |
| Hematocrit | 4.49% | 100% | Numerical |
| Blood platelet count | 2.79% | 1.81% | Numerical |
| Thrombin time | 5.07% | 100% | Numerical |
| International normalized ratio | 5.01% | 1.77% | Numerical |
| Prothrombin time | 5.01% | 1.69% | Numerical |
| Activated partial thromboplastin time | 5.06% | 1.73% | Numerical |
| Fibrinogen | 5.08% | 100% | Numerical |
| Albumin | 2.94% | 1.54% | Numerical |
| Globulin | 2.57% | 100% | Numerical |
| Albumin-globulin ratio | 2.57% | 100% | Numerical |
| Total protein | 2.57% | 100% | Numerical |
| Direct bilirubin | 2.57% | 100% | Numerical |
| Indirect bilirubin | 2.57% | 100% | Numerical |
| Total bilirubin | 2.57% | 1.86% | Numerical |
| Total bile acid | 3.46% | 100% | Numerical |
| ALT | 2.57% | 1.83% | Numerical |
| AST | 2.57% | 1.47% | Numerical |
| AST/ALT | 2.57% | 100% | Numerical |
| Alkaline phosphatase | 2.57% | 100% | Numerical |
| Glutamyl transpeptidase | 2.57% | 100% | Numerical |
| White blood cell count | 2.74% | 1.82% | Numerical |
| Monocyte percentage | 2.74% | 100% | Numerical |
| Monocyte count | 2.74% | 100% | Numerical |
| Lymphocyte percentage | 2.74% | 100% | Numerical |
| Lymphocyte count | 2.74% | 1.94% | Numerical |
| Neutrophil granulocyte percentage | 2.74% | 100% | Numerical |
| Neutrophil granulocyte count | 2.74% | 100% | Numerical |
| Acidophil percentage | 2.74% | 100% | Numerical |
| Acidophil count | 2.74% | 100% | Numerical |
| Basophil percentage | 2.89% | 100% | Numerical |
| Basophil count | 2.89% | 100% | Numerical |
| Creatinine | 2.90% | 1.73% | Numerical |
| Estimated glomerular filtration rate | 3.02% | 100% | Numerical |
| Serum cystatin C level | 2.54% | 100% | Numerical |
| Blood urea | 2.92% | 100% | Numerical |
| Uric acid | 2.90% | 100% | Numerical |
| Blood glucose | 2.92% | 4.12% | Numerical |
| Serum chlorine | 3.01% | 100% | Numerical |
| Serum sodium | 2.98% | 100% | Numerical |
| Serum potassium | 3.02% | 2.28% | Numerical |
| Serum phosphorus | 3.54% | 100% | Numerical |
| Serum calcium | 3.91% | 100% | Numerical |
| Serum magnesium | 3.91% | 100% | Numerical |
| Anion gap | 3.00% | 100% | Numerical |
| Cholesterol | 3.33% | 100% | Numerical |
| Triglyceride | 3.33% | 100% | Numerical |
| High density lipoprotein | 3.33% | 100% | Numerical |
| Low density lipoprotein | 3.33% | 100% | Numerical |
| Hydroxybutyrate dehydrogenase | 3.37% | 100% | Numerical |
| Creatine kinase | 3.36% | 100% | Numerical |
| Lactate dehydrogenase | 3.36% | 100% | Numerical |
| β-hydroxybutyrate | 5.28% | 100% | Numerical |

Footnote: Missing rate of 100% indicated that this variable was not included in the external dataset. Abbreviations: BMI, body mass index; ASA, American Society of Anesthesiologists; NYHA, New York Heart Association; COPD, chronic obstructive pulmonary disease; SpO_2_, oxygen saturation; CO_2_CP, carbon dioxide combining power; RDW-CV, coefficient of variation of red blood cell distribution width; RDW-SD, standard deviation of red blood cell distribution width; MCHC, mean corpuscular hemoglobin concentration; ALT, alanine aminotransferase; AST, aspartate aminotransferase.

Supplementary Table S2. Definitions of postoperative pulmonary complications.

| Complication | Definition |
| --- | --- |
| Unplanned mechanical ventilation | Unplanned requirement of non–invasive or invasive mechanical ventilation after surgery |
| Atelectasis | Lung opacification with a shift of the mediastinum, hilum or hemidiaphragm toward the affected area, and compensatory overinflation in the adjacent nonatelectatic lung (1) |
| Pulmonary congestion | Clinical signs of congestion, including dyspnoea, oedema, rales, and jugular venous distention, with or without chest x–ray demonstrating increase in vascular markings and diffuse alveolar interstitial infiltrates (2) |
| Respiratory infection | Need of antibiotics for a suspected infection and one or more of the following criteria: new or changed sputum, new or changed lung opacities, fever and/or white blood cell count > 12 × 10^9^ l^-1^ (3) |
| Pleural effusion | Chest radiograph demonstrating blunting of the costophrenic angle, Loss of sharp silhouette of the ipsilateral hemidiaphragm in upright position, evidence of displacement of adjacent anatomical structures or (in supine position) a hazy opacity in one hemithorax with preserved vascular shadows (4) |
| Pneumothorax | Air in the pleural space with no vascular bed surrounding the visceral pleura (5) |
| Respiratory failure | The presence of one of the following: postoperative PaO_2_<60 mmHg on room air; PaO_2_:FiO_2_ ratio<300 mmHg with oxygen therapy |

Footnote: Abbreviations: PaO_2_, partial pressure of oxygen in arterial blood; FiO_2_, inspired oxygen fraction.

References:

1. Duggan M, Kavanagh BP. Pulmonary atelectasis: A pathogenic perioperative entity. Anesthesiology (2005) 102:838–54. doi: 10.1097/00000542-200504000-00021

2. Karalapillai D, Weinberg L, Peyton P, Ellard L, Hu R, Pearce B, et al. Effect of Intraoperative Low Tidal Volume vs Conventional Tidal Volume on Postoperative Pulmonary Complications in Patients Undergoing Major Surgery: A Randomized Clinical Trial. JAMA (2020) 324:848-58. doi: 10.1001/jama.2020.12866

3. McAlister FA, Bertsch K, Man J, Bradley J, Jacka M. Incidence of and risk factors for pulmonary complications after nonthoracic surgery. Am J Respir Crit Care Med (2005) 171:514 –7. doi: 10.1164/rccm.200408-1069OC

4. Maskell NA, Butland RJ. Standards of Care Committee, British Thoracic Society: BTS guidelines for the investigation of a unilateral pleural effusion in adults. Thorax (2003) 58:ii8 –17. doi: 10.1136/thorax.58.suppl_2.ii8

5. Henry M, Arnold T, Harvey J. Standards of Care Committee, British Thoracic Society: BTS guidelines for the management of spontaneous pneumothorax. Thorax (2003) 58:ii39 –52. doi: 10.1136/thorax.58.suppl_2.ii39

Supplementary Table S3. Patients’ characteristics in the derivation dataset.

| Variable | Finding(n=12,240) |
| --- | --- |
| Male sex | 6909(56.4) |
| Age | 71(67-75) |
| Height, cm | 160(155-167) |
| Weight, kg | 60(54-67) |
| BMI | 23.4(21.3-25.6) |
| Smoking | 1079(8.8) |
| Alcohol | 334(2.7) |
| Systolic blood pressure | 134(122-146) |
| Diastolic blood pressure | 80(72-87) |
| Respiratory rate | 20(19-20) |
| Heart rate | 78(71-87) |
| Body temperature | 36.5(36.3-36.5) |
| ASA classification |  |
| I | 26(0.2) |
| Ⅱ | 6055(49.5) |
| Ⅲ | 6072(49.6) |
| Ⅳ | 85(0.7) |
| Ⅴ | 1 |
| Ⅵ | 1 |
| NYHA classification |  |
| I | 6746(55.1) |
| Ⅱ | 4876(39.8) |
| Ⅲ | 583(4.8) |
| Ⅳ | 35(0.3) |
| Functional capacity |  |
| >6MET | 1894(15.5) |
| 3-6MET | 7967(65.1) |
| <3MET | 2379(19.4) |
| Frailty assessment |  |
| Tiredness | 720(5.9) |
| Decreased endurance | 498(4.1) |
| Low activity | 475(3.9) |
| Comorbidities | 435(3.6) |
| Weight loss | 327(2.7) |
| General condition |  |
| Partial dependence | 3782(30.9) |
| Dependence | 207(1.7) |
| Conscious state |  |
| Sober | 12,212(99.8) |
| Somnolence | 19(0.2) |
| Lethargy | 1 |
| Light coma | 6(0.1) |
| Deep coma | 2 |
| Breath-holding test |  |
| ≥30s | 4172(34.1) |
| 20~29s | 7113(58.1) |
| 10~19s | 888(7.2) |
| <10s | 67(0.5) |
| Mallampati airway classification |  |
| I | 2175(17.8) |
| Ⅱ | 8987(73.4) |
| Ⅲ | 1010(8.2) |
| Ⅳ | 68(0.5) |
| Limited mouth opening | 191(1.6) |
| Mandibular protrusion test | 63(0.5) |
| Neck movement test | 63(0.5) |
| Emergent surgery | 121(1.0) |
| Open surgery | 7052(57.6) |
| Endoscopic surgery | 5230(42.7) |
| Endovascular surgery | 73(0.6) |
| Surgery type |  |
| Orthopaedics | 2293(18.7) |
| General surgery | 6071(49.6) |
| Thoracic surgery | 1167(9.5) |
| Cardiovascular surgery | 517(4.2) |
| Other | 2192(17.9) |
| Estimated operation time |  |
| <2h | 6645(54.3) |
| 2~4h | 4693(38.3) |
| ≥4h | 902(7.4) |
| Estimated blood loss |  |
| <10% estimated blood volume | 10,932(89.3) |
| 10~25% estimated blood volume | 1241(10.1) |
| >25% estimated blood volume | 67(0.6) |
| Comorbidities |  |
| Hypertension |  |
| I | 1458(11.9) |
| Ⅱ | 1915(15.7) |
| Ⅲ | 1196(9.8) |
| Diabetes mellitus |  |
| Non-insulin dependent | 1107(9.0) |
| Insulin dependent | 466(3.8) |
| Respiratory infection within last 1 month | 202(1.7) |
| COPD |  |
| Stable | 506(4.1) |
| Exacerbations | 13(0.1) |
| Repeated exacerbations within 1 year≥3 | 11(0.1) |
| Ddyspnea |  |
| I | 329(2.7) |
| Ⅱ | 426(3.5) |
| Ⅲ | 117(1.0) |
| Ⅳ | 36(0.3) |
| Difficult ventilation history | 42(0.3) |
| Difficult intubation history | 23(0.2) |
| Sleep apnea syndrome | 2787(22.8) |
| Airway obstruction | 135(1.1) |
| Stroke | 271(2.2) |
| Ischemic heart disease |  |
| Mild stenosis | 186(1.5) |
| Moderate stenosis | 107(0.9) |
| Severe stenosis | 67(0.6) |
| Valvular heart disease | 680(5.6) |
| Dilated cardiomyopathy | 22(0.2) |
| Hypertrophic cardiomyopathy | 16(0.1) |
| Congestive heart failure | 36(0.3) |
| Arrhythmia | 966(7.9) |
| Pericardial disease | 88(0.7) |
| Artery disease | 71(0.6) |
| Venous disease | 43(0.4) |
| Lower digestive tract hemorrhage within last 1 week | 88(0.7) |
| Upper digestive tract hemorrhage within last 1 week | 24(0.2) |
| Gastroesophageal reflux | 146(1.2) |
| Peptic ulcer | 171(1.4) |
| Psychiatric disorder | 88(0.7) |
| Neuromuscular disease | 64(0.5) |
| Thyroid dysfunction |  |
| Hyperthyroidism | 38(0.3) |
| Hypothyroidism | 156(1.3) |
| Autoimmune disease | 35(0.3) |
| Preoperative medical treatments |  |
| Vasoactive medications | 34(0.3) |
| Anti-heart failure drugs | 48(0.4) |
| Anticoagulants and antiplatelet agents | 447(3.7) |
| Glucocorticoid | 34(0.3) |
| Dialysis | 11(0.1) |
| Laboratory tests |  |
| SpO_2_, % | 98.0(96.6-99.0) |
| CO_2_CP, mmol/L | 24.2(22.4-26.0) |
| Hemoglobin, g/L | 128(114-139) |
| Red blood cell count, 10^12^/L | 4.2(3.7-4.6) |
| RDW-CV, % | 13.4(12.8-14.3) |
| RDW-SD, fL | 46.0(43.7-49.0) |
| Mean corpuscular hemoglobin, pg | 30.8(29.5-31.9) |
| MCHC, g/L | 327(320-334) |
| Mean corpuscular volume, fL | 93.8(90.6-97.0) |
| Hematocrit, L/L | 0.4(0.3-0.4) |
| Blood platelet count, 10^9^/L | 170(132-214) |
| Thrombin time, s | 17.9(17.3-18.7) |
| International normalized ratio | 0.9(0.9-1.0) |
| Prothrombin time, s | 10.8(10.3-11.4) |
| APTT, s | 27.2(25.7-29.0) |
| Fibrinogen, g/L | 2.9(2.5-3.5) |
| Albumin, g/L | 40.7(36.9-43.7) |
| Globulin, g/L | 25.2(22.5-28.2) |
| Albumin-globulin ratio | 1.6(1.4-1.8) |
| Total protein, g/L | 66.2(60.7-70.7) |
| Direct bilirubin, umol/L | 3.3(2.4-4.7) |
| Indirect bilirubin, umol/L | 8.0(6.0-10.7) |
| Total bilirubin, umol/L | 11.6(8.8-15.5) |
| Total bile acid, umol/L | 3.8(2.0-6.9) |
| AST, IU/L | 21(17-27) |
| ALT, IU/L | 16(12-25) |
| AST/ALT | 1.2(1.0-1.6) |
| Alkaline phosphatase, IU/L | 75(61-93) |
| Glutamyl transpeptidase, IU/L | 20(14-34) |
| White blood cell count, 10^9^/L | 6.1(4.9-7.9) |
| Monocyte percentage, % | 7.6(6.3-9.1) |
| Monocyte count, 10^9^/L | 0.5(0.3-0.6) |
| Lymphocyte percentage, % | 24.4(15.1-31.9) |
| Lymphocyte count, 10^9^/L | 1.4(1.0-1.8) |
| Neutrophil granulocyte percentage, % | 63.9(56.0-75.0) |
| Neutrophil granulocyte count, 10^9^/L | 3.8(2.8-5.6) |
| Acidophil percentage, % | 1.8(0.8-3.2) |
| Acidophil count, 10^9^/L | 0.1(0.1-0.2) |
| Basophil percentage, % | 0.5(0.3-0.7) |
| Basophil count, 10^9^/L | 0.03(0.02-0.04) |
| Creatinine, umol/L | 76(64-89) |
| e-GFR, ml/min/1.73m^2^ | 81.3(67.9-89.9) |
| Serum cystatin C level, mg/L | 1.0(0.9-1.2) |
| Blood urea, mmol/L | 5.4(4.4-6.6) |
| Uric acid, umol/L | 302(247-366) |
| Blood glucose, mmol/L | 5.4(4.8-6.5) |
| Serum chlorine, mmol/L | 104.0(102.0-105.9) |
| Serum sodium, mmol/L | 141.0(139.3-142.5) |
| Serum potassium, mmol/L | 4.0(3.8-4.3) |
| Serum phosphorus, mmol/L | 1.1(0.9-1.2) |
| Serum calcium, mmol/L | 2.2(2.1-2.3) |
| Serum magnesium, mmol/L | 0.9(0.8-0.9) |
| Anion gap, mmol/L | 16.7(14.8-18.4) |
| Cholesterol, mmol/L | 4.2(3.5-5.0) |
| Triglyceride, mmol/L | 1.1(0.8-1.6) |
| High density lipoprotein, mmol/L | 1.2(0.9-1.4) |
| Low density lipoprotein, mmol/L | 2.4(1.9-3.1) |
| Hydroxybutyrate dehydrogenase, IU/L | 130(114-150) |
| Creatine kinase, IU/L | 83(57-131) |
| Lactate dehydrogenase, IU/L | 170(149-196) |
| β-hydroxybutyrate, mmol/L | 0.1(0.1-0.2) |

Footnote: For numerical variables, data is presented in medians and interquartile ranges. For categorical variables, data is presented in number (percentage) of patients. Abbreviations: BMI, body mass index; ASA, American Society of Anesthesiologists; NYHA, New York Heart Association; MET, metabolic equivalent; COPD, chronic obstructive pulmonary disease; SpO_2_, oxygen saturation; CO_2_CP, carbon dioxide combining power; RDW-CV, coefficient of variation of red blood cell distribution width; RDW-SD, standard deviation of red blood cell distribution width; MCHC, mean corpuscular hemoglobin concentration; APTT, activated partial thromboplastin time; AST, aspartate aminotransferase; ALT, alanine aminotransferase; e-GFR, estimated glomerular filtration rate.

Supplementary Table S4. The number of patients in different risk groups divided by the deep neural network model.

| Risk group | Patients, n | % | PPCs, n | % | Rate, % |
| --- | --- | --- | --- | --- | --- |
| Low risk(≤0.3) | 9853 | 80.5 | 540 | 27.7 | 5.5 |
| Intermediate risk(0.3-0.7) | 1223 | 10.0 | 518 | 26.6 | 42.4 |
| High risk(>0.7) | 1164 | 9.5 | 891 | 45.7 | 76.6 |
| Total | 12240 | 100.0 | 1949 | 100.0 | 15.9 |

Footnote: Patients were divided into three groups reflecting low, intermediate, and high risk for PPCs based on the predicted probability assessed using the deep neural network model. Optimal cutoff values were confirmed by the minimum description length principle (low risk: ≤0.3, intermediate risk: 0.3< risk prediction value≤0.7, and high risk: >0.7). The result of chi-square test showed that the incidence of PPCs was significantly different between the three groups (P < 0.001). Abbreviations: PPCs, postoperative pulmonary complications.

Supplementary Table S5. Patients’ characteristics in the external dataset.

| Variable | Finding(n=7579) |
| --- | --- |
| Male sex | 4294(56.7) |
| Age | 70(67-75) |
| Height, cm | 160(155-167) |
| Weight, kg | 60(53-67) |
| BMI | 23(21-25) |
| Smoking | 756(10.0) |
| Alcohol | 159(2.1) |
| Systolic blood pressure | 135(122-147) |
| Diastolic blood pressure | 80(73-87) |
| Respiratory rate | 20(19-20) |
| Heart rate | 78(71-87) |
| Body temperature | 36.5(36.3-36.5) |
| ASA classification |  |
| I | 11(0.1) |
| Ⅱ | 3636(48.0) |
| Ⅲ | 3764(49.7) |
| Ⅳ | 129(1.7) |
| Ⅴ | 7(0.1) |
| Ⅵ | 1 |
| NYHA classification |  |
| I | 3645(48.1) |
| Ⅱ | 3442(45.4) |
| Ⅲ | 405(5.3) |
| Ⅳ | 28(0.4) |
| Functional capacity |  |
| >6MET | 580(7.7) |
| 3-6MET | 5539(73.1) |
| <3MET | 1337(17.6) |
| Frailty assessment |  |
| Tiredness | 484(6.4) |
| Decreased endurance | 397(5.2) |
| Low activity | 322(4.2) |
| Comorbidities | 274(3.6) |
| Weight loss | 224(3.0) |
| General condition |  |
| Partial dependence | 2357(31.1) |
| Dependence | 152(2.0) |
| Conscious state |  |
| Sober | 7526(99.3) |
| Somnolence | 35(0.5) |
| Lethargy | 4(0.1) |
| Light coma | 6(0.1) |
| Deep coma | 8(0.1) |
| Breath-holding test |  |
| ≥30s | 1280(16.9) |
| 20~29s | 5389(71.1) |
| 10~19s | 595(7.9) |
| <10s | 22(0.3) |
| Mallampati airway classification |  |
| I | 740(9.8) |
| Ⅱ | 6249(82.5) |
| Ⅲ | 566(7.5) |
| Ⅳ | 24(0.3) |
| Limited mouth opening | 33(0.4) |
| Mandibular protrusion test | 18(0.2) |
| Neck movement test | 33(0.4) |
| Emergent surgery | 393(5.2) |
| Open surgery | 3867(51.0) |
| Endoscopic surgery | 3646(48.1) |
| Endovascular surgery | 101(1.3) |
| Surgery type |  |
| Orthopaedics | 1438(19.0) |
| General surgery | 3334(44.0) |
| Thoracic surgery | 795(10.5) |
| Cardiovascular surgery | 292(3.9) |
| Other | 1680(22.2) |
| Estimated operation time |  |
| <2h | 3928(51.8) |
| 2~4h | 3082(40.7) |
| ≥4h | 516(6.8) |
| Estimated blood loss |  |
| <10% estimated blood volume | 6870(90.6) |
| 10~25% estimated blood volume | 624(8.2) |
| >25% estimated blood volume | 30(0.4) |
| Comorbidities |  |
| Hypertension |  |
| I | 997(13.2) |
| Ⅱ | 1166(15.4) |
| Ⅲ | 706(9.3) |
| Diabetes mellitus |  |
| Non-insulin dependent | 730(9.6) |
| Insulin dependent | 299(3.9) |
| Respiratory infection within last 1 month | 70(0.9) |
| COPD |  |
| Stable | 197(2.6) |
| Exacerbations | 8(0.1) |
| Repeated exacerbations within 1 year≥3 | 7(0.1) |
| Dyspnea |  |
| I | 184(2.4) |
| Ⅱ | 219(2.9) |
| Ⅲ | 42(0.6) |
| Ⅳ | 31(0.4) |
| Difficult ventilation history | 9(0.1) |
| Difficult intubation history | 11(0.1) |
| Sleep apnea syndrome | 1236(16.3) |
| Airway obstruction | 67(0.9) |
| Stroke | 164(2.2) |
| Ischemic heart disease |  |
| Mild stenosis | 255(3.4) |
| Moderate stenosis | 83(1.1) |
| Severe stenosis | 48(0.6) |
| Valvular heart disease | 426(5.6) |
| Dilated cardiomyopathy | 72(0.9) |
| Hypertrophic cardiomyopathy | 11(0.1) |
| Congestive heart failure | 16(0.2) |
| Arrhythmia | 463(6.1) |
| Pericardial disease | 79(1.0) |
| Artery disease | 42(0.6) |
| Venous disease | 36(0.5) |
| Lower digestive tract hemorrhage within last 1 week | 38(0.5) |
| Upper digestive tract hemorrhage within last 1 week | 20(0.3) |
| Gastroesophageal reflux | 77(1.0) |
| Peptic ulcer | 54(0.7) |
| Psychiatric disorder | 45(0.6) |
| Neuromuscular disease | 195(2.6) |
| Thyroid dysfunction |  |
| Hyperthyroidism | 22(0.3) |
| Hypothyroidism | 92(1.2) |
| Autoimmune disease | 34(0.4) |
| Preoperative medical treatments |  |
| Vasoactive medications | 14(0.2) |
| Anti-heart failure drugs | 23(0.3) |
| Anticoagulants and antiplatelet agents | 295(3.9) |
| Glucocorticoid | 26(0.3) |
| Dialysis | 14(0.2) |
| Laboratory tests |  |
| SpO_2_, % | 97(96-98) |
| Hemoglobin, g/L | 130(117-141) |
| Blood platelet count, 10^9^/L | 179(138-227) |
| Prothrombin time, s | 11.0(10.5-11.8) |
| International normalized ratio | 1.0(0.9-1.0) |
| APTT, s | 27.0(25.5-29.2) |
| Albumin, g/L | 41.8(38.9-44.5) |
| Total bilirubin, umol/L | 11.3(8.7-15.1) |
| Aspartate aminotransferase, IU/L | 20(17-26) |
| Alanine aminotransferase, IU/L | 16(12-24) |
| White blood cell count, 10^9^/L | 5.8(4.7-7.1) |
| Lymphocyte count, 10^9^/L | 1.5(1.1-1.8) |
| Creatinine, umol/L | 75(63-88) |
| Blood glucose, mmol/L | 5.3(4.8-6.2) |
| Serum potassium, mmol/L | 4.0(3.7-4.2) |

Footnote: For numerical variables, data is presented in medians and interquartile ranges. For categorical variables, data is presented in number (percentage) of patients. Abbreviations: BMI, body mass index; ASA, American Society of Anesthesiologists; NYHA, New York Heart Association; MET, metabolic equivalent; COPD, chronic obstructive pulmonary disease; SpO_2_, oxygen saturation; APTT, activated partial thromboplastin time.

Figure legend:

Supplementary Figure S1. Overall architecture of the deep neural network model.

This figure shows the overall architecture of the deep neural network model. The deep neural network model incorporates three different pathways: numerical data embedding, categorical data embedding, and free-text data embedding. Numerical variables were discretized in the Binning layer before embedding. In the free-text data embedding pathway, MedBERT was performed to acquire the contextualized word embeddings. These embeddings were then averaged by mean-pooling to generate the final free-text data embedding. The outputs from these three embedding pathways were concatenated vertically and fed into the Transformer layer. Multi-Head Attention was used to explore the relationships between the input embeddings and to estimate the importance of each variable. The final embedding was fed into the Multi-Layer Perceptron layer to obtain the prediction result.
